# A case of disseminated cryptococcus in an immunocompetent individual necessitating ventriculoperitoneal shunt

**DOI:** 10.1016/j.mmcr.2021.03.001

**Published:** 2021-03-17

**Authors:** Daniel Myers, Brooke McVaney, Zachary Higginbotham

**Affiliations:** a Northwestern University, Chicago, IL, USA; b West Virginia School of Osteopathic Medicine, Lewisburg, WV, USA; c Marshall School of Medicine Huntington, WV, USA

**Keywords:** Cryptococcus, Immunocompetent, Hepatitis C, Ventriculoperitoneal shunt, HIV

## Abstract

Cryptococcosis is a rare fungal disease causes primarily by two opportunistic organisms. It is usually seen in the immunocompromised but rarely it can infect immunocompetent individuals. We present a case of disseminated cryptococcus manifesting as cryptococcal meningitis in a young immunocompetent individual that led to substantial nonobstructive hydrocephalus which required VP shunt placement.

## 1 Introduction

**Fig. 1:**
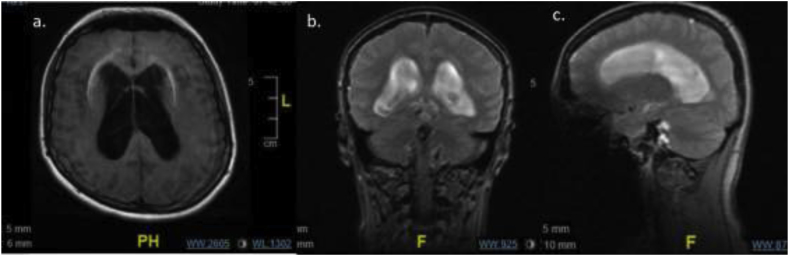
MRI on presentation (day 0), ventriculomegaly is seen on all three views: T1 flair axial (a), T2 coronal (b), T2 saggital (c). Lack of ventriculomegaly of the fourth ventricle and any obstructive mass is best visualized on saggital view (c).

Cryptococcosis meningoencephalitis is a severe fungal disease of the central nervous system. The disease commonly afflicts the immunocompromised, most notably those with HIV [1]. It is caused by infection from two distinct forms of encapsulated yeasts, Cryptococcus neoformans and Cryptococcus gattii. Infection occurs when the spores are inhaled and disseminated via the lungs to the bloodstream. Cryptococcosis has been estimated at over 500,000 cases per year with 1-year mortality rates that vary based on geographic location. From 20% in the US to as high as 70%. in sub-Saharan Africa [1]. There are other risk factors such as hematological malignancies, sarcoidosis, liver disease, idiopathic CD4^+^ lymphopenia, and immunotherapies. Rarely, *Cryptococcus* can produce disease in immunocompetent hosts [2]. More recently, there is emerging evidence that intravenous drug abuse (IVDA), even without HIV infection, can be a risk factor for cryptococcal disease [3]. IVDA is a well-known route of infection by hepatitis C, and this virus has been associated with coinfection with several infectious diseases [4]. Hepatitis C has been shown to cause immunosuppression via T-cell dysfunction, innate immunity dysregulation, and adaptive immunity exhaustion which may allow for disseminated cryptococcal infection in an apparent immunocompetent host [5]. In this report, we present a case of disseminated *Cryptococcus* in an immunocompetent host who was a known intravenous drug abuser with a history of chronic hepatitis C, who subsequently developed extensive nonobstructive hydrocephalus necessitating ventriculoperitoneal shunt placement. (see Fig. 1)

## 2 Case

A 23-year-old Caucasian female presented to the emergency room in July 2020 defined as day zero due to obtundation for approximately 2 hours. She had noted transient episodes of confusion and posterior headaches for 3–4 months prior that had been worsening in duration and frequency. Her medical history included anxiety, depression, and chronic hepatitis C for which she was antiviral therapy naive. The patient had a history of polydrug abuse in which she admitted to using IV heroin, methamphetamines and cocaine, as well as a tobacco smoking history of uncertain duration. The patient recently had returned from Oklahoma 2 weeks prior but noted no exposures to pigeons. Her vital signs were within normal limits and her physical exam was benign. Chest x-ray showed no abnormalities. Urine drug screen was positive for cannabinoids, opiates, and benzodiazepines. CT and MRI of the brain on day zero demonstrated enlargement of the 3rd and lateral ventricles with transependymal CSF absorption, but no significant enlargement of the 4th ventricle and no obstructing mass was identified (Fig. 1). EEG was normal. Lumbar puncture was performed and showed WBC 39mm^−3^ with lymphocytic predominance, RBC 16/μl, protein 129mg/dl, glucose <1 mg/dl, opening pressure >57 cm H2O (Table 1). A few encapsulated yeasts were seen on Gram stain. Meningitis PCR panel was positive for *Cryptococcus neoformans/gattii.* Serum cryptococcal antigen was positive and thus the patient was started on IV liposomal amphotericin B 200 mg/day and oral flucytosine 1000 mg every 6 hours and was admitted to the Internal Medicine service on day 1. HIV-1/2 antibody and p24 antigen testing was negative and CD4^+^ count was 502. HCV antibody was positive with HCV quantitative RNA value of 3.3 million copies, genotype 1a with a fibrosis score of F0. Serial LPs were performed to decrease the opening pressure and treat the hydrocephalus with caution for herniation. Serial LPs showed an opening pressure of 57 cm H_2_O and 55 cm H_2_O on day four and five respectively. As there was little reduction in pressure, a lumbar drain was placed on hospital day 10, draining 10 cc/hour for the first 24 hours of use. For the subsequent 9 days, 10 cc were drained every 2 hours. Clinically, she remained stable during all the LPs. The lumbar drain also subsequently failed to reduce the opening pressure sufficiently (opening pressure of 55 cm H_2_O on day 14), and therefore the neurosurgery team decided to perform ventriculoperitoneal shunting on hospital day 20 which induced rapid clinical improvement. The patient was discharged on oral fluconazole 800 mg daily on hospital day 29 and was eventually lost to follow up.

**Table 1:** CSF Results throughout admission.

Lumbar Puncture Results	Day 3	Day 17	Day 26	Reference Range
Color	Colorless	Colorless	Colorless	
Turbidity	Clear	Clear	Clear	
White Blood Cell	39/mm^3^	55/mm^3^	13/mm^3^	0-5/mm^3^
Red Blood Cell	0/mm^3^	13mm^3^	16mm^3^	<1/mm^3^
Neutrophil	71%	7%	2%	2–6%
Lymphocyte	24%	82%	8%	40–80%
Monocyte	4%	10%	3%	15–45%
Protein	113 mg/dl	91 mg/dl	129 mg/dl	20–40 mg/dL
Glucose	7 mg/dl	2 mg/dl	<1 mg/dl	45–80 mg/dl

## 3 Discussion

Invasive cryptococcal disease (cryptococcosis) is caused either by *C. neoformans* or *C. gattii*. These fungal species are found throughout the world and is commonly in association with pigeon feces, amoeba, sowbugs, and in the hollows of some tree species [1]. The organisms can cause disease by two mechanisms which include latency and reactivation or primary acquisition [1].

*Cryptococcus neoformans* meningoencephalitis is a rare cause of disease in immunocompetent individuals [6]. The pathogenicity of the organism is not fully understood but its polysaccharide glucuronoxylomannan capsule (GXM), phospholipase activity, and extracellular vesicle upregulation allows for virulence factor production, host adaption and immune protection [7]. During acute infection, the hosts complement system begins the response and is induced by the GXM capsule which leads to alveolar macrophage phagocytosis where the fungus can replicate intracellularly. Macrophages secrete cytokines and induce a Th1 response leading to the involvement of CD4^+^ and CD8^+^ cells which assist in recruitment and direction of immune cells and cell-mediated destruction [8]. This supports the disease association with states of immunodeficiency such as malignancy, CD4^+^ lymphopenia and notably, AIDS where T-cells are depleted. It is increasingly recognized that states of subclinical immunodeficiency can also precipitate disease which primarily include, but are not limited to, uncontrolled diabetes, alcoholism, chronic infection, sarcoidosis, IVDA, and chronic liver disease [8]. In our patient, it was thought that a combination of her history of intravenous drug abuse and history of chronic Hepatitis C with high active viral load led to a subclinical decrease in immunocompetence and subsequent infection with *Cryptococcus*.

Serum from chronically HCV-infected patients displayed a lower level of C5b-9 and a reduced antimicrobial effect on model organisms compared to healthy volunteers. Also, liver biopsy in infected patients show reduce C9 mRNA expression [9]. Hepatitis C also has two known structural proteins, 3/4 A (NS3/4A) and non-structural 5A (NS5A), which both contribute to immune dysregulation and reduced host response by cleaving toll like receptors and disrupting cytokine signaling. Together, providing a hypothetical mechanism that supports opportunistic infection in HCV [10]. El-Serag et al. found an association between HCV infection and multiple other infectious diseases and concluded that patients suffering from HCV infection had a significantly higher prevalence of cryptococcal infections (0.4% vs 0.1%), compared to controls [4]. Our case highlights the possible contribution of HCV in cryptococcal infection, but also that clinicians may consider screening for HCV in cryptococcosis, especially in those with a substance abuse history.

We also consider that her injection drug use may have contributed to her infection. It is uncertain how disseminated cryptococcal disease might develop in patients who use intravenous drugs. However, methamphetamine has been shown to promote dissemination of cryptococcus from the respiratory tract into the CNS in animal models by altering phagocytosis and disrupting antigen presentation on certain immune cells [11]. Furthermore, methamphetamine accumulation in the lungs can results in defects in macrophages and neutrophils, creating conditions more likely to result in disease. And finally, methamphetamine exposure causes *C.* *neoformans* to modulate its capsule which may be advantageous during infection [11].

Elevated intracranial pressure (ICP) in patients with HIV-associated cryptococcal meningitis is associated with an increased risk for mortality and prompt management is necessary [12]. Several treatment options exist for managing elevated intracranial pressure including intermittent CSF drainage by means of sequential lumbar punctures, insertion of a lumbar drain, or placement of a ventriculoperitoneal shunt. Medical approaches, including the use of corticosteroids, acetazolamide, or mannitol, have not been shown to be effective in the setting of cryptococcal meningitis [12]. In cases where repeated lumbar punctures or use of a lumbar drain fail to control elevated pressure symptoms, or when persistent or progressive neurological deficits are present, a ventriculoperitoneal shunt is indicated [12]. HIV negative and positive cases are managed the same although the data is sparse on outcomes in HIV-negative associated cryptococcal intracranial hypertension (ICH). Liu et al. proposed early placement of a shunt is helpful in decreasing uncontrollable elevated opening pressure regardless if ventriculomegaly was present in non-HIV cryptococcosis patients. The shunt could not only relieve the symptoms of ICH but could also improve the clinical features of these patients. Therefore, early diagnosis and use of a ventriculoperitoneal shunt before the onset of severe neurological deficit symptoms, could be beneficial and essential [13].

In summary, this case highlights the possible important role of social and medical factors that can contribute to non-HIV associated cryptococcal dissemination and the need for prompt recognition, diagnosis, and treatment of ICH in these patients.

## Funding source

None.

## Consent

Written informed consent was obtained from the patient or legal guardian(s) for publication of this case report and accompanying images. A copy of the written consent is available for review by the Editor-in-Chief of this journal on request.

## Declaration of competing interest

No conflict of interest declared.

## References

[1] Maziarz E.K., Perfect J.R. (2016 Mar). Cryptococcosis. Infect Dis Clin North Am..

[2] Lui G., Lee N., Ip M., Choi K.W., Tso Y.K., Lam E., Chau S., Lai R., Cockram C.S. (2006 Mar). Cryptococcosis in apparently immunocompetent patients. QJM.

[3] O'Meara T.R., Alspaugh J.A. (2012). The Cryptococcus neoformans capsule: a sword and a shield. Clin. Microbiol. Rev..

[4] El-Serag H.B., Anand B., Richardson P., Rabeneck L. (2003 Jan). Association between hepatitis C infection and other infectious diseases: a case for targeted screening?. Am. J. Gastroenterol..

[5] Missale G., Cariani E., Ferrari C. (2004 Nov). Role of viral and host factors in HCV persistence: which lesson for therapeutic and preventive strategies?. Dig. Liver Dis..

[6] Mada P., Nowack B., Cady B., Joel Chandranesan A.S. (2017 Jul 18). Disseminated cryptococcosis in an immunocompetent patient. BMJ Case Rep..

[7] Alspaugh J.A. (2015 May). Virulence mechanisms and Cryptococcus neoformans pathogenesis. Fungal Genet. Biol..

[8] Rohatgi S., Pirofski L.A. (2015). Host immunity to Cryptococcus neoformans. Future Microbiol..

[9] Millman A.J., Nelson N.P., Vellozzi C. (2017 Jun). Hepatitis C: review of the epidemiology, clinical care, and continued challenges in the direct acting antiviral era. Curr Epidemiol Rep.

[10] Imran M., Waheed Y., Manzoor S., Bilal M., Ashraf W., Ali M., Ashraf M. (2012 Jun 22). Interaction of Hepatitis C virus proteins with pattern recognition receptors. Virol. J..

[11] Patel D., Desai G.M., Frases S., Cordero R.J., DeLeon-Rodriguez C.M., Eugenin E.A., Nosanchuk J.D., Martinez L.R. (2013 Jul 30). Methamphetamine enhances Cryptococcus neoformans pulmonary infection and dissemination to the brain. mBio.

[12] Fessler R.D., Sobel J., Guyot L., Crane L., Vazquez J., Szuba M.J., Diaz F.G. (1998 Feb 1). Management of elevated intracranial pressure in patients with Cryptococcal meningitis. J. Acquir. Immune Defic. Syndr. Hum. Retrovirol..

[13] Liu J., Chen Z.L., Li M., Chen C., Yi H., Xu L., Tan F., Peng F.H. (2018 May 1). Ventriculoperitoneal shunts in non-HIV cryptococcal meningitis. BMC Neurol..

